# Annotations

**Published:** 1897-10-09

**Authors:** 


### ANNOTATIONS.

### The Mothers of England.

In commenting on a case in which a woman died in childbirth, in consequence of the ignorance of the midwife in attendance, the *Lancet* has hit upon the exact truth in regard to the midwives question. It says: "Whatever views may be entertained on the question of a Midwives Bill, one thing is becoming clearer every day—that midwives, without any necessary acquaintance with the dangers of childbirth, or the most elementary principles on which they should be met, cannot be allowed to continue their work unchecked. Women of the poorer classes must somehow be protected from such an obvious risk to life." The natural thing to suggest as a cure for the state of affairs brought to light at the inquest on the case reported, was that a Midwives Bill should be passed; and this was the view expressed by the coroner. What we would point out, however, is that no Midwives Bill that has so far been brought before Parliament would prevent the most ignorant of women from attending and taking pay for attending as many confinements as they like. These Bills protect the name of midwife, but impose no restriction on any woman, however ignorant, doing the midwife's work. This is their weak spot. We believe with the *Lancet*, that these women "cannot be allowed to continue their work unchecked." That is just what we have contended for all along, and it is because the Bills so far brought before Parliament have not even attempted to interfere with their *work* that we have objected to them as inadequate, and as not giving the required protection to the mothers of England.

### The Degeneration of Monthly Reviews.

The great Professor Mommsen is reported once to have said, referring to a well-known Orientalist domiciled in England, "And haf you no hombocks in your own country, zat you most get zem from us?" We might apply this equally to the importation of quacks from that go-ahead country, the United States, where there is established, we believe, a quack manufacturing company capable of supplying the commodity in unlimited quantity. The modern quack no longer appeals to the populace from the market place—he aims at higher game. His victims are the well-to-do, his agora the public press. Till lately he traded in electricity; but, alas! the long arm of the law cut short many promising careers in this department. Now a far more pernicious "treatment" is being skilfully advertised, more pernicious in that while "electricity" affected merely the pocket, the new panacea is calculated to ruin the health as well. This latest abomination is known as the "Salisbury treatment," and an anonymous article in the *Westminster Review* for August, for the opinions expressed in which the editors of that admirable periodical explicitly decline to be responsible, pungently illustrates the objects and methods of those who profit by it. This "treatment" appears to have been imported afresh into England by Mrs. Elma Stuart, whom the article devotes itself to advertising, even to the extent of giving her address. It is, of course, primarily intended for the prolongation "of the gift of life bestowed upon God's creatures," and a suggestion is made that, although the originator of the "cure" has amassed "mere wealth," his disciples desire for him "public recognition" as well! The essential principle of the treatment is that all disease arises from the stomach—with one or two trivial exceptions. To quote the *Westminster* advertisement: "Heart disease, paralysis, consumption, diabetes, Bright's disease, rheumatism, rheumatic gout, gout, asthma, tumours, neuralgia, dropsy, pleurisy—they are all treated of (in Mrs. Stuart's book), and quite scientifically shown to be but the natural, inevitable result of impaired digestive faculties in the first stage of their appearance." The sad part of this sort of thing is that it actually seems to attract and influence certain of the laity, who like to diagnose their own cases and play tricks with their constitution. That under certain rare circumstances a beef-steak diet may be of use was known to medical men long before Dr. Salisbury began those microscopical and chemical studies which have brought him in so much money, but its indiscriminate employment by ignorant persons has far more than counterbalanced its occasional utility. It is, of course, quite in the proper order of things that Mrs. Stuart should herself be a sufferer cured by the "treatment" in question, and that her great desire should be to benefit "God's creatures." There would be nothing to be surprised at if we saw it in the advertising columns of a religious weekly. Still we are a little surprised at finding it in the body of the *Westminster Review*. Someone must have taken a great deal of trouble to get such an advertisement inserted in such a place, and if they find themselves rewarded for it we can but condole with the editor of that periodical on the mental calibre of his clientèle. Not that we can hold him free from blame. He may only have been trying a cynical experiment in the hope of discovering by what sort of people he was read, but we think he should have exercised a more careful guardianship over the weaker members of his flock.

### Chinese Laundrymen.

That the cheap labour of the Chinese is not regarded as an altogether unmixed blessing by the inhabitants of the United States is well known, but how nasty are some of its accompaniments is perhaps not so generally recognised. Both as cooks and as laundrymen they are much employed, and we have heard many dwellers on the Pacific coast say they could not get on without them. Still, they have little tricks and dodges which, however well they may serve their purpose, are not nice, and certainly, from a sanitary point of view, do not come up to modern standards. One of these pleasing methods is what is termed the mouth spray. An English laundrymaid damps the linen by sprinkling it with water. This, however, involves putting down the iron. But John Chinaman knows a better way. Filling his mouth with water, he squirts it on the linen in front of the iron, and by long practice he is able to do this with much nicety—save the mark! blowing it out in the form of a fine spray just where it is required. Some people are found to object to this simple and easy labour-saving device, and we see it stated in the *Medical News* of New York that an inspection of the Chinese laundries has been ordered by the Health Commissioner of St. Louis, "who fears that some may mouth-spray their work with tubercle bacilli, and thus spread contagion." The inspection of Chinamen is, however, by no means an easy task.

